# Pulmonary Pleomorphic Carcinoma Mimicking Small Bowel Obstruction

**DOI:** 10.7759/cureus.109097

**Published:** 2026-05-18

**Authors:** Arjun N Bhatt, Bhavini Singh, Raag Patel, Michael Toscano, Jayanth Keshavamurthy

**Affiliations:** 1 Ophthalmology, Augusta University Medical College of Georgia, Augusta, USA; 2 Obstetrics and Gynecology, Augusta University Medical College of Georgia, Augusta, USA; 3 Pulmonology, Augusta University Medical College of Georgia, Augusta, USA; 4 Pathology, Augusta University Medical College of Georgia, Augusta, USA; 5 Radiology, Augusta University Medical College of Georgia, Augusta, USA

**Keywords:** pulmonary pleomorphic carcinoma, pulmonary sarcomatoid carcinoma (psc), small bowel obstruction, spindle-shaped cells, squamous cell lung carcinoma

## Abstract

Pulmonary pleomorphic carcinoma (PC) is a rare and aggressive subtype of sarcomatoid carcinoma. It is characterized by poorly differentiated cells with both epithelial and mesenchymal features and often presents as a peripheral lung mass. Metastasis to the gastrointestinal tract is exceedingly rare. We report the unusual case of an 87-year-old man with a prior history of lung adenocarcinoma who presented with abdominal pain secondary to a small bowel obstruction. Imaging revealed a right lower quadrant mass, and surgical exploration uncovered an undifferentiated malignant tumor involving the small bowel mesentery. Histopathological analysis demonstrated spindle cells, giant cells, and rhabdoid features with positive immunostaining for thyroid transcription factor-1 (TTF-1), Napsin A, and CK7, consistent with metastatic PC. Additional imaging revealed widespread disease including masses in the gastrohepatic region, adrenal gland, and sacrum. This case highlights a rare manifestation of pulmonary PC with metastasis to the small bowel causing obstruction, an uncommon but important differential diagnosis in lung cancer patients presenting with abdominal symptoms. Early recognition is critical, as atypical metastatic patterns may delay diagnosis and impact clinical management.

## Introduction

Pulmonary pleomorphic carcinoma (PC) is a rare, highly malignant subtype of sarcomatoid carcinoma, with an incidence of 0.1-0.4% of all lung cancers. Even in early-stage disease, PC demonstrates a propensity for vascular invasion, present in 57% of pN0 cases, and high postoperative recurrence rates of 54%, with 50% of recurrences occurring within six months of resection [1,2]. Per the World Health Organization (WHO) 2021 Classification of Thoracic Tumors, PC is a subtype of sarcomatoid carcinoma, defined as a poorly differentiated non-small cell lung cancer (NSCLC) containing at least 10% spindle and/or giant cells or consisting entirely of these elements. It frequently contains conventional NSCLC components, most commonly adenocarcinoma or squamous cell carcinoma, admixed with sarcomatoid elements, with the ≥10% threshold distinguishing it from other NSCLC subtypes [3]. PC carries a poor prognosis, with a median overall survival of nine months and five-year survival rates of 23-25% across all stages [4]. The tumor demonstrates significant resistance to conventional platinum-based chemotherapy, and surgical resection remains the primary treatment for localized disease [4]. Here, we discuss a case of metastatic PC presenting as small bowel obstruction.

## Case presentation

An 87-year-old man with a history of adenocarcinoma, chronic obstructive pulmonary disease (COPD), hypertension, hyperlipidemia, diabetes, chronic kidney disease, and a significant smoking history presented with abdominal pain for three days. In the week prior, he experienced loose stools, diarrhea, and excessive flatulence. Computed tomography (CT) imaging of the abdomen and pelvis obtained during admission revealed small bowel obstruction caused by a right lower quadrant abdominal mass measuring 7×8 cm, as well as a large 10 cm mass in the gastrohepatic region, an enlarging adrenal mass, and a lytic lesion in the sacrum (Figure 1). He subsequently underwent an exploratory laparotomy with resection. Pathology demonstrated an undifferentiated malignant tumor composed of sheets of spindle cells, giant cells, and scattered small cells with rhabdoid/signet-ring cell morphology. Numerous mitotic figures and areas of necrosis were present. The tumor was centered in the mesentery with extension to the muscularis propria. Histopathology confirmed tumor infiltration into the small bowel wall on hematoxylin and eosin (H&E) staining at low magnification (Figure 2A) and pleomorphic tumor cells with spindle morphology and visible mitotic figures at higher magnification (Figure 2B). Immunohistochemical staining demonstrated diffuse nuclear positivity for keratin AE1/AE3, CK7, Napsin A, as well as thyroid transcription factor-1 (TTF-1) (Figure 3), supporting pulmonary origin. Taken together, the patient's prior history of lung adenocarcinoma, along with the morphologic and immunohistochemical profile, supported the diagnosis of metastatic pulmonary PC. Given the extent of disease, patient age, and overall performance status, a goals of care discussion was held with the patient and family following diagnosis. The patient was transitioned to comfort-focused care and survived approximately two months following the diagnosis of metastatic pulmonary PC, consistent with the poor prognosis reported in the literature.

**Figure 1:**
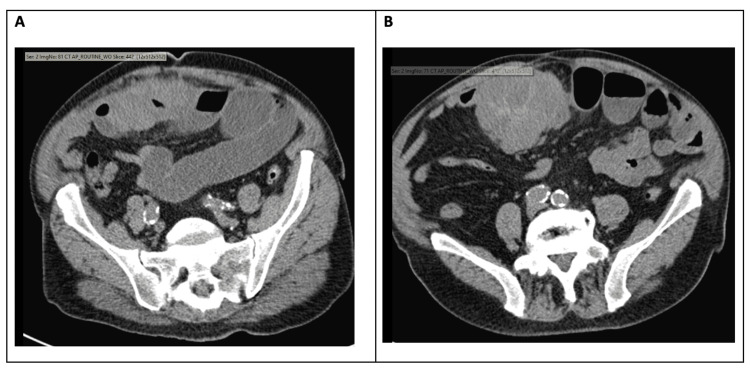
Axial computed tomography of the abdomen/pelvis Axial computed tomography of the abdomen/pelvis obtained during admission: (A) small bowel obstruction secondary to a right lower quadrant abdominal mass measuring 7×8 cm and (B) a 10 cm gastrohepatic mass with associated adrenal enlargement and a lytic lesion in the sacrum.

**Figure 2:**
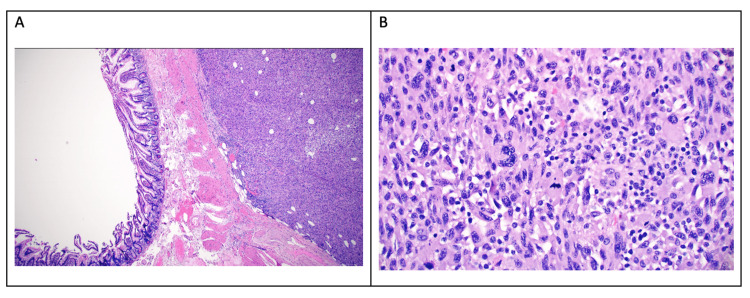
Histology of tumor infiltration at low magnification Histopathological findings: (A) hematoxylin and eosin stain at 4× magnification showing the tumor infiltrating the small bowel wall and (B) hematoxylin and eosin stain at 40× magnification demonstrating pleomorphic tumor cells with spindle morphology and visible mitotic figures.

**Figure 3:**
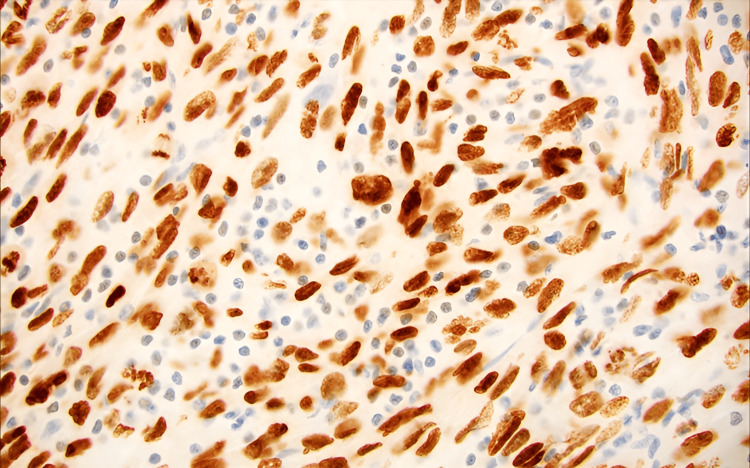
Immunohistochemical stain Immunohistochemical staining for thyroid transcription factor-1 demonstrating diffuse nuclear positivity in tumor cells.

## Discussion

Pulmonary PC is a rare and highly malignant variant of sarcomatoid carcinoma, accounting for only a small fraction of lung cancers [5]. It is defined histologically by spindle and giant cells with epithelial elements, most often adenocarcinoma or squamous cell carcinoma. Pulmonary PC typically presents in older male smokers as a large, peripheral lung mass with frequent necrosis [5]. The prognosis is poor due to rapid progression, early metastasis, and resistance to systemic therapy. Surgical resection remains the mainstay of treatment for localized disease, but relapse is common and post-relapse survival is often measured in months [4,6].

Reported rates of gastrointestinal metastasis from primary lung cancer vary across studies, ranging from 0.2-1.7% in some series to 4.7-14% in others, and carry a poor prognosis with a median survival of 2-6 months following identification [7,8]. One review examining 44 unique case reports of primary lung cancer metastasizing to the colon found that 45% were attributable to squamous cell carcinoma, 11% to small cell carcinoma, and 7% to large cell carcinoma [9]. Notably, among PC subtypes, giant cell carcinoma has a recognized propensity for small bowel metastasis, and clinicians should maintain a heightened index of suspicion for gastrointestinal involvement in patients with this histological subtype presenting with abdominal symptoms [10]. Gastrointestinal involvement most often presents with nonspecific abdominal pain, obstruction, or bleeding, and imaging findings can closely mimic primary gastrointestinal malignancies, making differentiation challenging. In our patient, bowel obstruction was the presenting manifestation of metastatic pulmonary PC.

Immunohistochemistry is indispensable for establishing pulmonary origin, especially in cases with ambiguous clinical and radiologic findings. Markers such as TTF-1 and Napsin A are highly specific for lung adenocarcinomas and are routinely used to distinguish metastatic pulmonary tumors from primary gastrointestinal cancers [11]. This profile was present in our case and, together with the patient's history of lung adenocarcinoma, supported metastatic pulmonary PC. On imaging, pulmonary PC typically manifests as a large peripheral lung mass, often exceeding 5 cm, with heterogeneous enhancement and areas of necrosis on CT. Fluorodeoxyglucose-positron emission tomography (FDG-PET) characteristically demonstrates high metabolic avidity; however, these findings are not specific enough to establish a definitive diagnosis and can overlap with other primary lung malignancies and metastatic lesions. In our case, chest CT was not obtained, and the diagnosis relied on abdominal CT findings in conjunction with histopathological and immunohistochemical analysis [12].

Systemic chemotherapy, particularly platinum-based regimens, has shown limited benefit in pulmonary PC, with most cases demonstrating resistance [13]. Emerging evidence suggests that immune checkpoint inhibitors may offer some benefit in select patients, especially those with high PD-L1 expression, but response rates remain modest and durable remissions are uncommon [14]. Due to the rarity and heterogeneity of this tumor, no standardized treatment algorithm exists, and management is typically individualized based on performance status, disease extent, and symptom control. In our patient, given his advanced age, poor performance status, and widespread metastatic disease at presentation, active systemic therapy was not pursued, and goals of care were redirected toward comfort-focused management, consistent with current evidence supporting individualized decision-making in this setting.

The relationship between the patient's prior lung adenocarcinoma and the subsequent diagnosis of pulmonary PC warrants consideration. Three mechanisms may explain this finding. First, sarcomatoid transformation, in which conventional adenocarcinoma undergoes epithelial-mesenchymal transition to acquire sarcomatoid features, is a recognized phenomenon in NSCLC [15]. Second, sampling bias at the time of the original biopsy may have led to the underrepresentation of sarcomatoid elements; as PC requires a ≥10% sarcomatoid threshold for diagnosis, small biopsy specimens may fail to capture these components, resulting in an initial diagnosis of pure adenocarcinoma [16]. Third, the PC may represent a second primary tumor independent of the prior adenocarcinoma, a plausible scenario given the patient's significant smoking history [17]. Detailed records from the patient's prior lung adenocarcinoma diagnosis were unavailable for review, precluding comparison of prior and current pathological specimens and definitive characterization of the relationship between the two diagnoses. Chest CT was not obtained during this admission, limiting the assessment of the primary pulmonary lesion. Future similar cases would benefit from chest CT imaging and comprehensive molecular profiling, including estimated glomerular filtration rate (EGFR), KRAS, MET exon 14, and PD-L1 status, both of which would further strengthen diagnostic certainty and inform treatment decision-making.

## Conclusions

Pulmonary PC is a highly malignant tumor with a recognized potential for gastrointestinal metastasis. The diagnostic challenge in such cases arises not from histomorphological features alone but from the atypical clinical presentation of this patient, which may delay the recognition and workup of a pulmonary primary. Despite its relative infrequency, clinicians should consider the possibility of gastrointestinal metastasis when evaluating lung cancer patients exhibiting signs of abdominal pain. This case underscores the importance of maintaining a broad differential diagnosis in patients with a history of lung cancer who present with nonspecific abdominal complaints. While outcomes are often poor, timely recognition of unusual metastatic patterns can prevent delays in diagnosis and guide appropriate management.
